# Supplementary figures and images for: IL-17A exacerbates psoriasis in a STAT3 overexpressing mouse model
Source: PeerJ. 2023 Jul 14;11:e15727. doi: 10.7717/peerj.15727 (PMC10351506; doi:10.7717/peerj.15727)

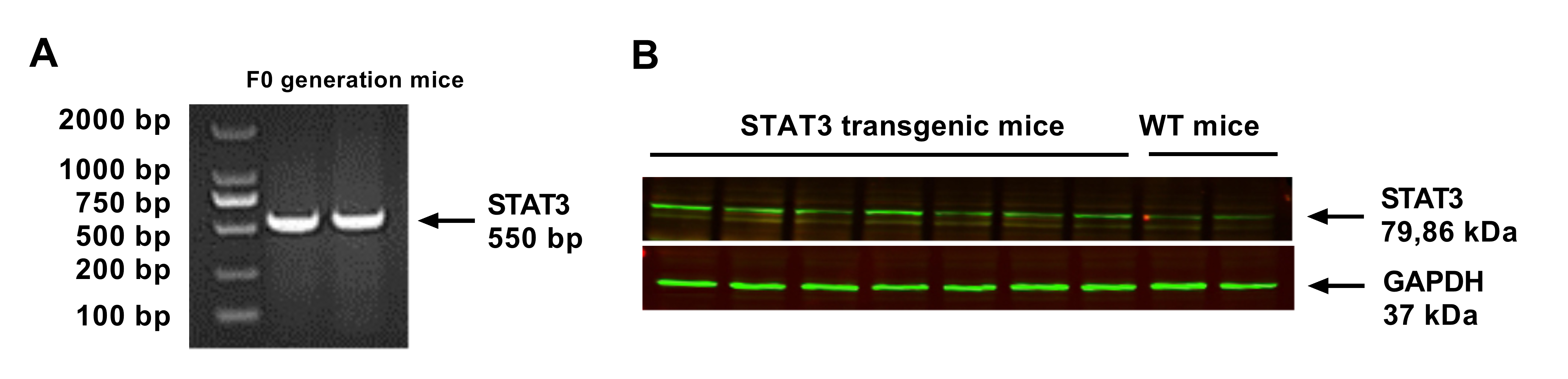

Supplement: Supplemental Information 1 — (A) STAT3 gene expression from the tails of transgenic mice (F0) were measured by RT-PCR. (B) Protein expression from the dorsal skin of STAT3 mice were measured by Western blot. [file peerj-11-15727-s001.png]

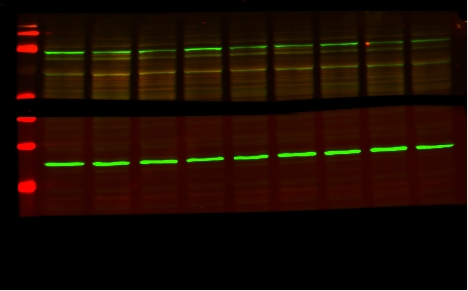

Supplement: Supplemental Information 2 [file peerj-11-15727-s002.jpg]
