# Supplementary material for: IL-17A exacerbates psoriasis in a STAT3 overexpressing mouse model
Source: PeerJ. 2023 Jul 14;11:e15727. doi: 10.7717/peerj.15727 (PMC10351506; doi:10.7717/peerj.15727)
Supplement: Supplemental Information 3 [file peerj-11-15727-s003.docx]

**Supplementary Table:**

Primer sequences for qPCR validation

| **Name** | **Forward primer sequence (5’-3’)** | **Reverse primer sequence (5’-3’)** | **length** | **RefSeq ID** |
| --- | --- | --- | --- | --- |
| s100A8 | ATGCCCTCTACAAGAATGACTTC | GAGATGCCACACCCACTTTTAT | 168bp | NM_013650.2 |
| s100A9 | ACAAATGGTGGAAGCACAGTT | ATCAGCATCATACACTCCTCAAAG | 135bp | NM_009114.3 |
| Sprr2e | TGTCTTACCAACAGCAGCAGTG | CATTTCTGCTGGTATGAGGGAG | 172 bp | NM_011471.2 |
| Sprr2g | TGTCCTCCTCCAAAGTGTCCT | CCCCTTGTTCTGAAGATTGCT | 154bp | NR_003548.1 |
| Sprr2d | TCCTGAGAATCCAGCACTATGTC | GACAAGGCTCTGGGCATTT | 181 bp | NM_011470.2 |
| LCE3d | CCAGCAGAGCCAGAAGCA | TGTAGCACAGCAGGAAGAGG | 121 bp | NM_001270436.1 |
| LCE3e | CAGCCTCCTCCCAAGTGC | AGACTGCTGACCACTGCCAC | 225 bp | NM_001254725.1 |
| LCE3f | TGCTGAGATGTTTCCCAAGAGT | CACCCAGGCAGTTATCAAAAG | 105 bp | NM_001018079.1 |
| TNF-α | TAGCCAGGAGGGAGAACAGA | TTTTCTGGAGGGAGATGTGG | 127bp | NM_013693.3 |
| IL1-β | GAAGAAGAGCCCATCCTCTG | TCATCTCGGAGCCTGTAGTG | 98bp | NM_008361.4 |
| CXCL1 | TGTTGTGCGAAAAGAAGTGC | TACAAACACAGCCTCCCACA | 91bp | NM_008176.3 |
| CXCL2 | AAGTTTGCCTTGACCCTGAA | AGGCACATCAGGTACGATCC | 180 bp | NM_009140.2 |
| CCL3 | TACAAGCAGCAGCGAGTACC | GAGCAAAGGCTGCTGGTTTC | 246bp | NM_011337.2 |
| CCL4 | ACCAATGGGCTCTGACCCTC | CTTGGAGCAAAGACTGCTGGT | 109bp | NM_013652.2 |
| CD14 | CTCTGTCCTTAAAGCGGCTTAC | GTTGCGGAGGTTCAAGATGTT | 191 bp | NM_009841.4 |
| TLR7 | ATGTGGACACGGAAGAGACAA | GGTAAGGGTAAGATTGGTGGTG | 207bp | NM_001290755.1 |
| β-actin | GGTCATCACTATTGGCAACG | TCCATACCCAAGAAGGAAGG | 72bp | NM_007393.5 |
